# Education and Message Framing Increase Willingness to Undergo Research Lumbar Puncture: A Randomized Controlled Trial

**DOI:** 10.3389/fmed.2020.00493

**Published:** 2020-09-18

**Authors:** Megan G. Witbracht, Olivia M. Bernstein, Vanessa Lin, Christian R. Salazar, S. Ahmad Sajjadi, Dan Hoang, Chelsea G. Cox, Daniel L. Gillen, Joshua D. Grill

**Affiliations:** ^1^Institute for Memory Impairments and Neurological Disorders, University of California, Irvine, Irvine, CA, United States; ^2^Department of Statistics, University of California, Irvine, Irvine, CA, United States; ^3^Graduate Medical Sciences, Boston University School of Medicine, Boston, MA, United States; ^4^Department of Neurology, University of California, Irvine, Irvine, CA, United States; ^5^Department of Psychiatry and Human Behavior, University of California, Irvine, Irvine, CA, United States; ^6^Department of Neurobiology and Behavior, University of California, Irvine, Irvine, CA, United States; ^7^Institute for Clinical and Translational Science, University of California, Irvine, Irvine, CA, United States

**Keywords:** message framing, cerebrospinal fluid, biomarker, recruitment, lumbar puncture

## Abstract

Reluctance to undergo lumbar puncture (LP) is a barrier to neurological disease biomarker research. We assessed whether an educational intervention increased willingness to consider research LP and whether message framing modified intervention effectiveness. We randomly assigned 851 recruitment registry enrollees who had previously indicated they were unwilling to be contacted about studies requiring LP to gain or loss framed video educational interventions describing the procedure and the probability of experiencing adverse events. The gain framed intervention emphasized the proportion of individuals free of adverse events; the loss frame emphasized the proportion experiencing adverse events. The primary outcome for the study was the participant's post-intervention agreement to be contacted about studies requiring LP. Participants were mean (SD) age 60.1 years (15.7), 69% female (*n* = 591), and mostly college educated and white. Among the 699 participants who completed the study, 43% (95% CI: 0.39, 0.47; *n* = 301) changed their response to agree to be contacted about studies requiring LP. We estimated that participants randomized to the gain framed intervention had 67% higher odds of changing their response compared to those randomized to the loss frame (Odds Ratio = 1.67; 95% CI: 1.24, 2.26; *p* < 0.001). A classification and regression tree model identified participants' pre-intervention willingness as the strongest predictor of changing response. Education, in particular education that alerts participants to the probability of not experiencing adverse events, may be an effective tool to increase participation rates in research requiring LP.

## Introduction

Alzheimer's disease (AD) is an incurable neurodegenerative disease marked by progressive cognitive and functional deterioration. The pathological hallmarks of AD are accumulation in the brain of neuritic plaques and neurofibrillary tangles. Assays of amyloid beta, phosphorylated tau, and total tau protein concentrations in cerebrospinal fluid (CSF) support AD diagnosis, predict AD neuropathology, and are valuable biomarkers in AD research (1). Recent discoveries reveal AD CSF changes may precede symptom onset and biomarker characterization of the “preclinical” phases of AD is an active area of research (2).

CSF is obtained through lumbar puncture (LP). Adverse events associated with the procedure range from backache to postural headache and occur in around 20% of research LPs in most studies (3–7). Post-LP headache frequency is dependent upon the equipment and technique used, and demographic factors of the person undergoing the LP (3, 8, 9). For example, atraumatic needles, gravity drip, and older participant age are associated with lower risk. Despite its safety profile, apprehension to undergo LP is a common barrier to biomarker research participation (10, 11). Methods to increase participation in research requiring LP could accelerate discovery and reduce selection bias, since the procedure is often optional and agreement may be associated with specific demographic variables (e.g., white race and high education) (10, 11).

Public health campaigns have borrowed from cognitive psychology in efforts to encourage healthy and altruistic decision-making. Presenting equivalent information with specific positive or negative connotations, referred to as message framing, has affected group rates of specific decisions and enabled the development of optimal health educational interventions (12). To our knowledge, message framing has not been examined in the context of recruiting healthy volunteers to biomarker research. We performed a randomized study to test the hypothesis that an educational intervention and specific message framing to communicate equivalent quantitative information on LP-associated risks would increase willingness to be contacted for research involving LP among enrollees in a recruitment registry.

## Materials and Methods

### Design

We conducted a single-blind, parallel-group, randomized controlled trial to examine whether educational interventions using gain and loss frames differed in effectiveness for increasing willingness to consider studies involving LP. We administered the study exclusively online. We used YouTube, a video sharing platform, to deliver the video intervention and Research Electronic Data Capture (REDCap), an online database management software, to collect study data (13). Upon clicking a link in an invitation email, participants were asked two pre-intervention questions, watched an educational video, and then were asked several post intervention questions.

### Participants

Participants were recruited from the University of California at Irvine Consent-to-Contact (C2C) Registry, a local, online research recruitment registry (14). Adults age 18 years and older who had enrolled in the C2C Registry and had indicated that they were not willing to be contacted about studies requiring LP were eligible to participate. At the time the study was initiated, 57% of the 3,970 subjects (*n* = 2,263) enrolled in the registry fell into this category and were thus invited to participate. No other exclusion criteria were applied.

### Ethics

The Institutional Review Board at the University of California at Irvine (UCI) approved this study. A waiver of written consent was granted; consent was confirmed by active participation. Subjects were recruited by email, which included a description of the study and instructions for completing the study online. Any participant who received the email was eligible for a drawing for a $100 gift card. The email description did not disclose the design of the study; it explained that the purpose of the study was to better understand attitudes toward LP as a research procedure. Registrants were informed that participation would include watching several videos and answering questions.

### Intervention

The online educational intervention consisted of narrated PowerPoint slideshows loaded onto YouTube [Video 1: https://youtu.be/GMjomqJAnMg; Video 2: https://youtu.be/jzff427XPmc; Video 3: https://youtu.be/XOdjI9d70zs; Video 4a (gain frame): https://youtu.be/NB-qGaApre0; Video 4b (loss frame): https://youtu.be/CMhKeCUCDAg]. The videos lasted approximately 5 min and included information about the purpose of the research LP and the importance of CSF assays in the context of AD research. Videos described the LP procedure, the recommendations for post-procedure care, the frequency of LPs or similar procedures performed in a clinical setting, the qualifications of clinicians who perform the LP, and the frequency of adverse events associated with the procedure. Videos concluded with a discussion of the post-dural puncture headache, addressed concerns about perceived pain, and the improbability of paralysis. Questions to check for comprehension were embedded in the video.

**Figure 1:**
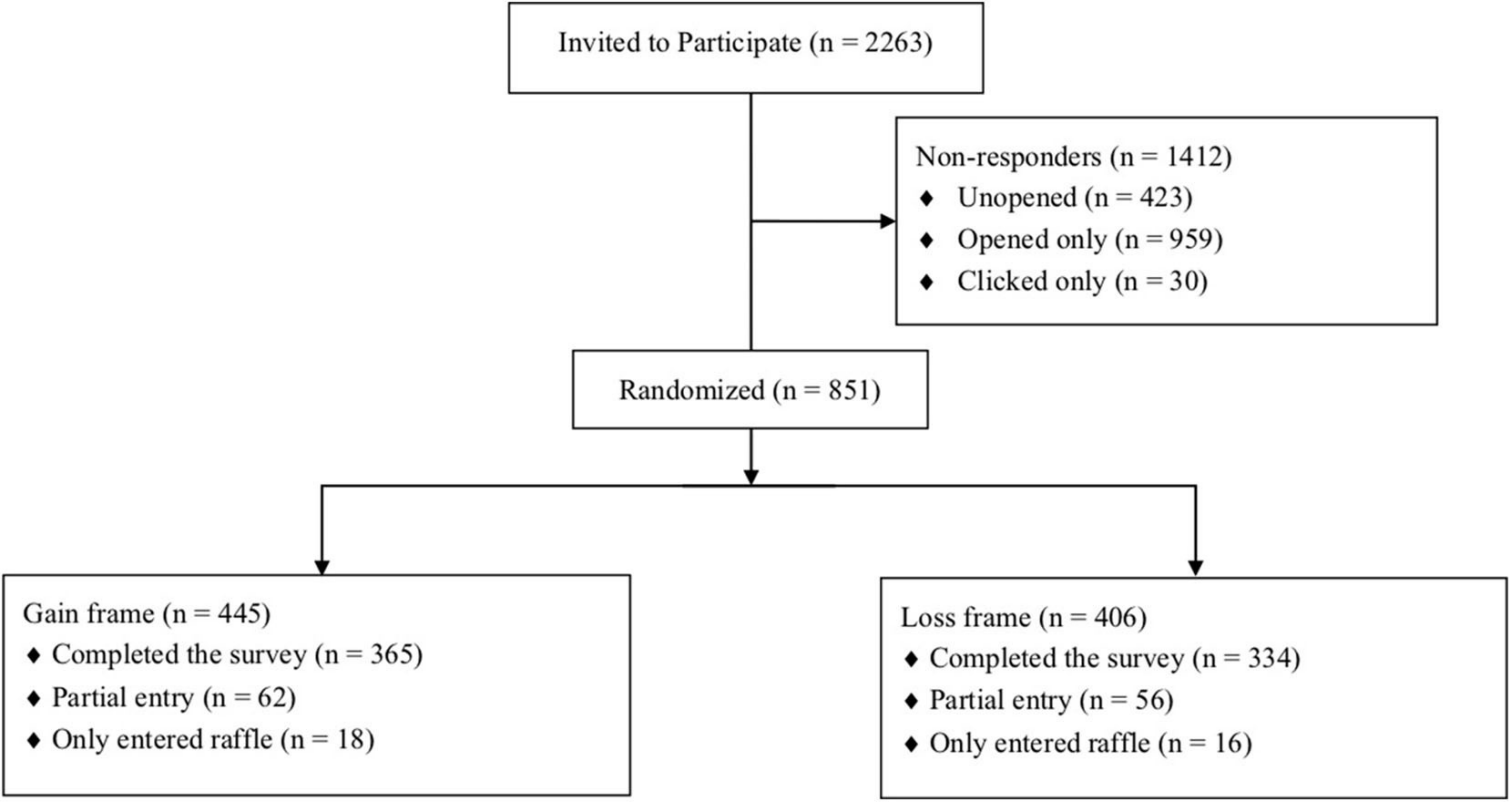
Attrition flow diagram showing the disposition of participants.

Participants were randomized to a gain or loss framed presentation of adverse event information. Randomization was performed through REDCap using a Javascript function. Adverse event frequency rates were extracted from Peskind et al. (5) and presented in multiple ways (as percentages, fractions, and with pictographs) to account for potential differences in numeracy within the sample (15). The framed interventions were balanced for slide number, intervention length, and used the same narrator and PowerPoint style (Figure 1). The loss framed intervention emphasized the frequency of adverse events; the gain framed intervention emphasized the number individuals free of the most common adverse events. The loss framed intervention was titled “LP Risks;” the gain framed intervention was titled “LP Safety.” The loss frame pictographs illustrated the number experiencing AEs in red (compared to white figures for the proportion not experiencing adverse events); the gain frame illustrated the number not experiencing adverse events in green (compared to white figures for the proportion who did experience adverse events) (16). The empirical information provided to participants in the loss framed and gained framed arms was equivalent, only differing in framing of the information. Participants were blinded to randomized design of the study and their assignment.

### Data Collection

Demographic information, medical history, and family history of AD were self-reported when enrolling in the registry (14). Participants also completed validated instruments assessing subjective cognitive function [the Cognitive Function Instrument (CFI)] (17) and research attitudes [Research Attitude Questionnaire (RAQ)] (18) when enrolling in the registry. Scores for the CFI range from 0 to 14, with higher scores indicating greater subjective complaints. Scores for the RAQ range from 7 to 35, with higher scores indicating more positive attitudes toward research.

After randomization but prior to the intervention, we further assessed participants' willingness to undergo a research LP with the following question: “*You indicated in the C2C Registry that you were not willing to hear about studies that involve a lumbar puncture. Today, how likely are you to undergo a lumbar puncture?*” Participants indicated their willingness using a 6-point Likert scale (from “extremely likely” to “extremely unlikely”). We assessed participants' perceived risk associated with the LP pre- and post-intervention with the following question: “*How risky do you consider the lumbar puncture procedure to be?*” Participants indicated their perceived risk using a 5-point Likert scale ranging from “not at all risky” to “extremely risky.” We asked participants to respond YES or NO to the following post-intervention question, “*When you submitted information about yourself in the C2C Registry, you indicated that you would not want to be contacted about studies that involve a lumbar puncture. At this time would you like us to change your answer in the C2C Registry so that you can be contacted for studies that include a lumbar puncture?*” The response to this question served as the study primary outcome.

### Statistical Analyses

Descriptive statistics summarized subject characteristics according to randomized groups at baseline. Ages were not provided from the registry for participants age >90 years; we assigned these four subjects an age of 90 for all analyses. *Post-hoc* sensitivity analyses to assess the impact of imputing age values up to 100 years revealed no qualitative differences from the study results presented here. To assess whether framing had an effect on change in agreement to be contacted for LP studies, we used a logistic regression model to estimate the odds ratio (OR) for change in agreement comparing gain frame to loss frame. The corresponding Wald-based 95% confidence interval (CI) and associated *p*-value for testing the null hypothesis of no framing effect were also computed.

We hypothesized that the effect of framing would differ by participants' pre-intervention willingness to undergo an LP. Willingness was re-categorized *a priori* into 3 groups for analysis. Participants who responded “extremely unlikely” were categorized as unlikely, participants who responded “moderately unlikely,” “slightly unlikely,” and “slightly likely” were categorized as neutral, and those who responded “moderately likely” and “extremely likely” were categorized as likely. We used a logistic regression model that included an interaction between pre-intervention willingness group and an indicator for randomized group to estimate the odds ratio for change in agreement, comparing gain frame to loss frame within each of the three willingness groups. To test whether the effect of framing differed by pre-intervention willingness, we performed a likelihood ratio test (LRT) comparing nested models with and without interaction terms.

There is some evidence to support a role for perceived risk in mediating the effect of framing on behavior change (19). To test for potential mediation, we estimated the primary model with and without adjustment for post-intervention perceived risk in a *post-hoc* analysis and assessed the degree of attenuation of the main effect.

To identify subpopulations most likely to switch responses, we built a Classification and Regression Tree (CART) model that inherently includes interactions. Our model considered splitting the data on the following variables: intervention frame, age, sex, ethnicity, race, recruitment method (how subjects were recruited into the C2C Registry), number of comorbidities, number of concomitant medications, family history of AD, past neurological diagnosis, education level, CFI score, RAQ score, pre-intervention willingness, pre-intervention perceived risk, and the availability of a study partner. We used 10-fold cross-validation for estimation of out-of-sample classification performance. All tests were 2-sided and analyses were performed using R 3.6.0.

## Results

### Participants

As depicted in Figure 1, 851 (38%) of 2,263 eligible subjects enrolled in the study. Among enrollees, *n* = 445 were randomized to the positive frame and *n* = 406 to the negative frame. More than 80% of enrollees (*n* = 699) completed the study, including the post-intervention questionnaire. Table 1 describes the overall distribution of participant characteristics, stratified by randomized group. There were no apparent differences between the randomized groups. Subjects had a mean overall age of 60 years, most were female, college educated and white (Table 1). Prior to the educational intervention, most participants were “unlikely” (*n* = 326; 47%) or “neutral” (*n* =303; 43%) in their willingness to undergo LP (Table 1). Greater than 70% (*n* = 497) of participants reported that the LP was extremely, very or moderately risky.

**Table 1:** Baseline characteristics of randomized subjects stratified by framing group.

	**Loss frame (*n* = 406)**	**Gain frame (*n* = 445)**	**Total (*n* = 851)**
Age (years), mean (±SD; *n* missing)	59.2 (16.4; 4)	60.9 (15.0; 5)	60.1 (15.7; 9)
CFI Score, mean (±SD; *n* missing)	2.5 (2.6; 0)	2.6 (2.5; 0)	2.5 (2.5; 0)
RAQ, mean (±SD; *n* missing)	28.3 (4.3; 5)	28.3 (4.6; 0)	28.3 (4.5; 5)
Female sex, *n* (%)	291 (72)	297 (67)	588 (69)
**Race**			
White, *n* (%)	348 (86)	389 (87)	737 (87)
Asian, *n* (%)	21 (5)	22 (5)	43 (5)
Black or African American, *n* (%)	4 (1)	6 (1)	10 (1)
American Indian or Alaska Native, *n* (%)	3 (1)	0 (0)	3 (0)
Native Hawaiian or Other Pacific Islander, *n* (%)	0 (0)	2 (0)	2 (0)
Multiracial, *n* (%)	14 (3)	8 (2)	22 (3)
Other, *n* (%)	12 (3)	7 (2)	19 (2)
Refuse, *n* (%)	4 (1)	11 (2)	15 (2)
Latino ethnicity, *n* (%)	28 (7)	38 (9)	66 (8)
**Recruitment method**			
Email, *n* (%)	249 (61)	269 (60)	518 (61)
Community talk, *n* (%)	21 (5)	30 (7)	51 (6)
Postcard, *n* (%)	29 (7)	33 (7)	62 (7)
Other, *n* (%)	106 (26)	111 (25)	217 (25)
Missing, *n* (%)	1 (0)	2 (0)	3 (0)
**Education level**			
High School, *n* (%)	19 (5)	26 (6)	45 (5)
Some College or Trade School, *n* (%)	69 (17)	73 (16)	142 (17)
College or Higher, *n* (%)	315 (78)	341 (77)	656 (77)
Missing, *n* (%)	3 (1)	5 (1)	8 (1)
**Number of comorbidities**			
0, *n* (%)	20 (5)	20 (4)	40 (5)
1, *n* (%)	209 (51)	225 (51)	434 (51)
2+, *n* (%)	176 (43)	200 (45)	376 (44)
Missing, *n* (%)	1 (0)	0 (0)	1 (0)
**Concomitant medications**			
None	76 (19)	86 (19)	162 (19)
1–2, *n* (%)	130 (32)	151 (34)	281 (33)
3–4, *n* (%)	103 (25)	99 (22)	202 (24)
5+, *n* (%)	94 (23)	104 (23)	198 (23)
Missing, *n* (%)	3 (1)	5 (1)	8 (1)
Past neurological diagnosis, *n* (%)	53 (13)	59 (13)	112 (13)
Family history of AD, *n* (%)	51 (13)	49 (11)	100 (12)
Pre-intervention willingness to undergo LP^*^			
Unlikely, *n* (%)	157 (47)	169 (46)	326 (47)
Neutral, *n* (%)	151 (45)	152 (42)	303 (43)
Likely, *n* (%)	26 (8)	44 (12)	70 (10)
Pre-intervention perceived risk^*^			
Somewhat or not at all, *n* (%)	91 (27%)	111 (30%)	202 (29%)
Extremely, very or moderately, *n* (%)	243 (73%)	254 (70%)	497 (71%)

* *Pre-intervention quantities are reported for the 699 participants who completed the study (n = 334 in the loss frame and n = 365 in the gain frame)*.

### Intervention Effectiveness

Overall, 43% (95% CI: 0.39, 0.47; *n* = 301) of participants changed their response to agreement to be contacted about studies involving LP. Forty-nine percent of participants in the gain frame group (95% CI: 0.44, 0.54; *n* = 179), compared to 37% in the loss frame group (95% CI: 0.31, 0.42; *n* = 122), changed their response. Participants exposed to the gain frame were estimated to have a 67% (OR: 1.67 95% CI: 1.24, 2.26; *p* < 0.0001) higher odds of changing their response to allow contact about studies requiring LP, compared to those exposed to the loss frame.

**Figure 2:**
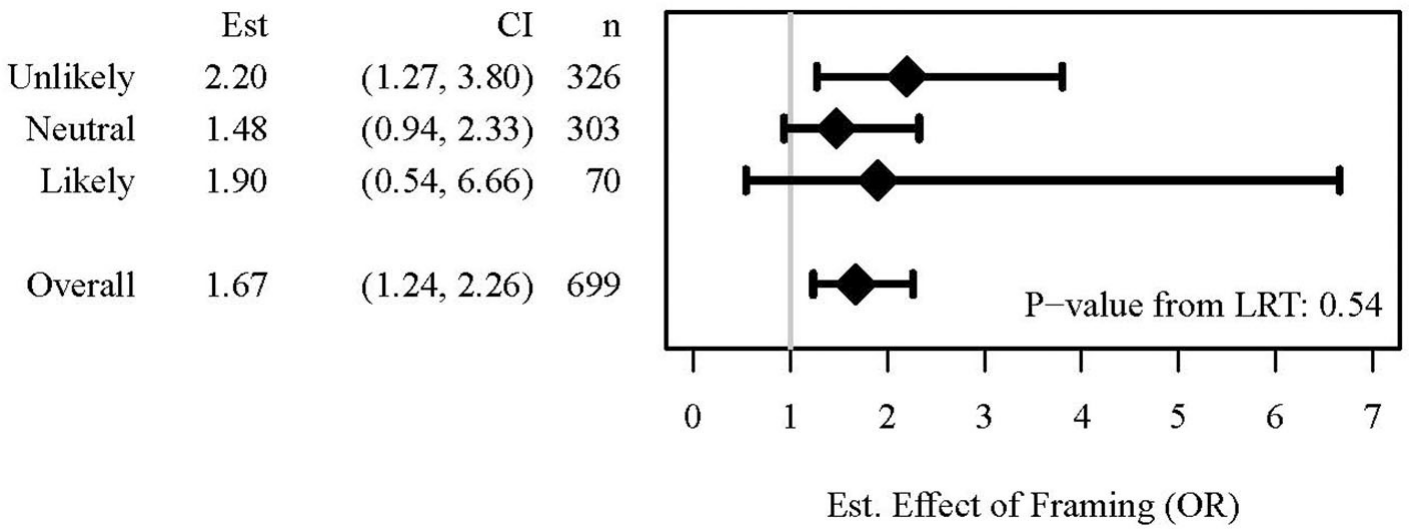
Forest Plot depicting the odds ratios (OR) for the effect of framing by pre-intervention willingness groups and the likelihood ratio test (LRT) to assess whether the effect of framing differed by pre-intervention willingness.

In secondary analyses (Figure 2), we did not find evidence that the effect of framing on change in agreement varied by pre-intervention willingness (LRT *p*-value for interaction = 0.541). Among subjects who indicated that they were unlikely to undergo LP prior to educational intervention, those who were exposed to the gain frame were estimated to have a 2.2-fold higher odds of changing their response compared to their counterparts exposed to the loss frame (OR: 2.20; 95% CI: 1.27, 3.80). Among those neutral or likely to undergo LP, we estimated that exposure to the gain frame was associated with 48% (OR: 1.48; 95% CI: 0.94, 2.33) and 90% (OR: 1.90; 95% CI: 0.54, 6.66) higher odds of changing their response, respectively, compared to the loss frame.

In exploratory analyses, we found that the frequency of rating the risk associated with having an LP as “extremely risky,” “very risky” or “moderately risky” decreased from 71% pre-intervention (*n* = 497) to 27% post-intervention (*n* = 191). Post-intervention, 24% (*n* = 86) and 31% (*n* = 105) of participants in the gain and loss frame groups, respectively, reported that they perceived the LP as extremely, very, or moderately risky. This explained some but not all of the effect of framing on changing agreement. Participants randomized to the gain framed intervention were estimated to have 57% higher odds of changing their response when compared to those randomized to the loss frame who had a similar perceived risk (OR: 1.57; 95% CI: 1.14, 2.15; *p* = 0.005).

**Figure 3:**
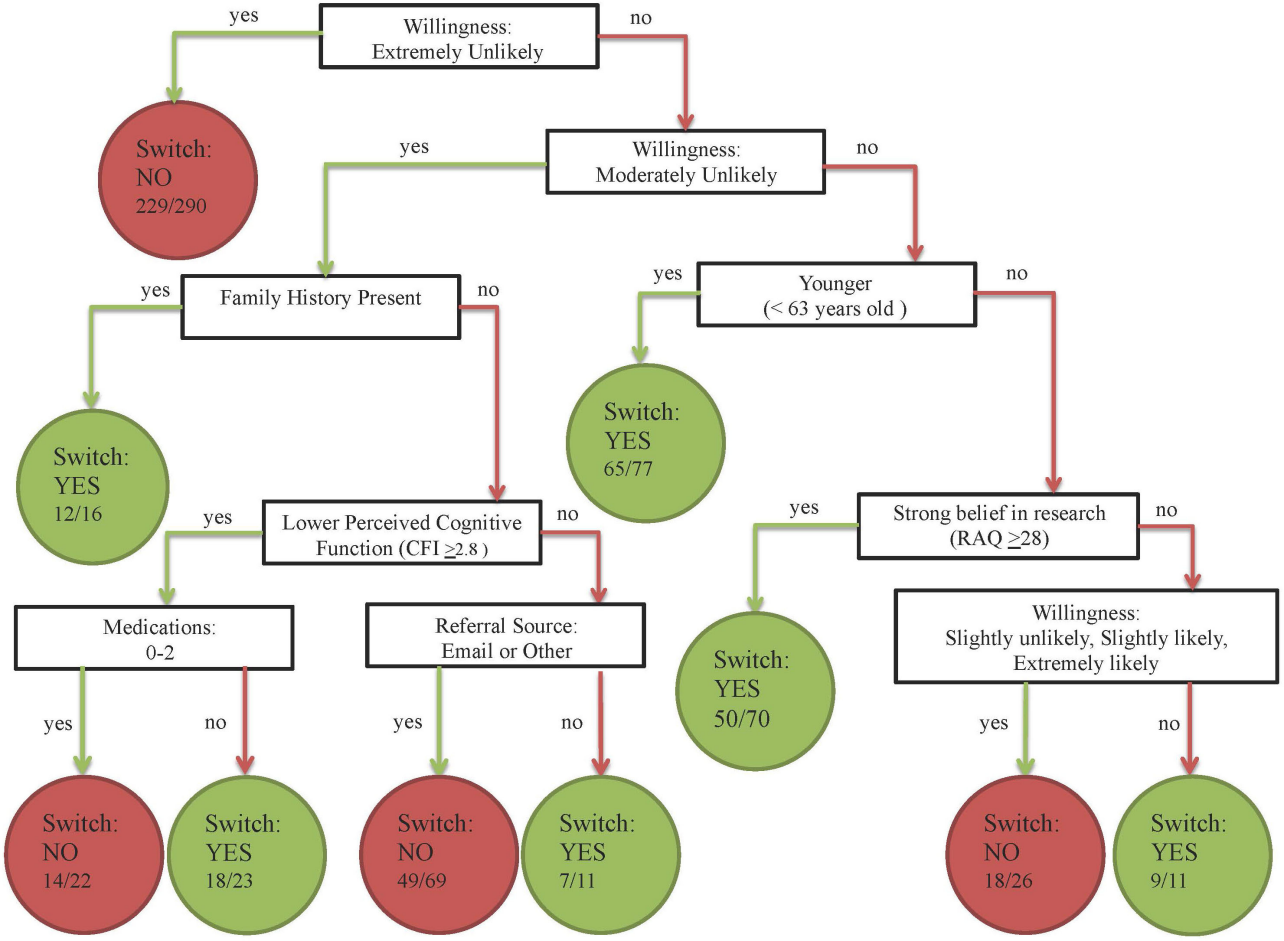
Illustration of Classification and Regression Tree (CART) for the subject characteristics most likely to predict change in response to agreement to be contacted for studies involving LP. The model considered splitting on the following variables: intervention frame, age, sex, ethnicity, race, referral method (how participants were recruited into the C2C Registry), number of comorbidities, number of concomitant medications, family history of AD, past neurological diagnosis, education level, CFI score, RAQ score, pre-intervention willingness, pre-intervention perceived risk, and the availability of a study partner. The nodes of the tree represent the splitting variables and the termini or leaves represent the predicted response and the proportion of subjects who responded in that direction.

An exploratory CART model identified seven predictors of the probability of changing one's response to agreement to be contacted for studies involving LP. Identified predictors included pre-intervention willingness, family history, age, RAQ score, CFI score, referral source, and medication use (Figure 3). The first two splits in the tree (accounting for the most variability in change in agreement) were from the pre-intervention willingness question. Of the 290 subjects who reported they were extremely unlikely to consider an LP pre-intervention, 229 did not change their response post-intervention. Among subjects who were moderately unlikely to consider an LP, those with a positive family history of AD were most likely to switch their response (12/16 subjects).

## Discussion

While it is widely regarded as a clinically safe procedure, the requirement to undergo LP is considered a significant barrier to AD research recruitment (20). Improvements in equipment and technique have reduced risk of pain and post-dural puncture headaches, yet lingering negative attitudes toward the procedure continue to impede CSF research. For example, while 4 in every 5 enrollees of our C2C Registry express willingness to be contacted for studies involving positron emission tomography imaging (and 9 in every 10 for magnetic resonance imaging), less than half agree to be contacted for studies involving LP (14). In this study, we found that 43% of these registry enrollees who completed an educational intervention changed their response to be willing to consider research involving LP. Furthermore, we discovered that incorporating gain framed education, emphasizing the proportion of participants who do not experience adverse events, resulted in a higher proportion of participants changing their response, compared to those who received an otherwise identical version emphasizing the proportion of participants who do experience adverse events. These findings provide new evidence that may instruct recruitment strategies to improve participation in AD biomarker research.

A recent study found that a poor understanding of the LP was associated with negative attitudes about the procedure (21). Interventions designed specifically to demystify the LP and to address post procedure side-effects may normalize the procedure and improve enrollment rates. Video guides may be particularly effective tools in increasing comprehension of the LP (22, 23). Our online educational video intervention decreased perceived risk and increased willingness to consider participation in studies involving LP, regardless of which framing technique was employed. Our intervention was also fully automated (i.e., the video was sent via email and viewed online), potentially reducing the faculty and staff requirements of education.

Message frame significantly impacted the effectiveness of the intervention. Our results indicate that for every eight subjects re-approached to consider LP, gain-framing elicited a change in response in one additional participant, compared to the loss frame. This equates to an estimated 12.5% increase in potential enrollment when incorporating the gain frame in large scale recruitments. Message framing has been examined extensively in the context of the promotion of health behaviors. Gain frame messages may be more effective in changing preventative behaviors, where risk is minimal and outcomes more certain (e.g., sunscreen use to reduce skin cancer risk); whereas loss frame messages are more effective in motivating detection type behaviors where risk is inherent and associated with disease occurrence and incurring difficult consequences (e.g., mammograms for the detection of breast cancer) (12). Unlike health promoting behaviors, research participation in healthy populations often involves accepting a varying degree of risk with few if any personal benefits. Few published reports have examined the effects of message framing to motivate research enrollment decisions, although at least some studies find that gain frame messaging increases willingness to enroll and to make other altruistic decisions (24, 25).

Our study utilized a specific type of framing known as *attribute framing*, which evaluates a single quality of an object and is distinct from the more complex valance of *goal framing* originally proposed by Kahneman and Tversky (26, 27). Gain attribute framing may offer advantage for dichotomous research enrollment decisions by creating positive associations with the item being framed. In our study, the gain frame message may have promoted the storage of neutral or positive information about the LP, instead of negative risk information. In support of this theory, we observed that perceived risk assessed post-intervention partially explained the framing effect on LP willingness.

Like previous studies (28), these results suggest that the manner in which quantitatively equivalent information is delivered can affect decision-making. Could this have ethical implications to informed consent? To be ethical, informed consent must be free of coercion and undue influence, and the participant must have the capacity and adequate information to make a voluntary and autonomous choice (29). The two arms of this study were offered alternate representations of the same information [the proportion of a group anticipated to experience (or not experience) adverse events] and the choices made in both groups were voluntary. Is one of these presentations ethically preferable to the other? Did the positive frame lead to irrational decisions by some (30, 31)? Or might the positive frame have allowed participants to assess the choice from a different perspective, one that more frequently opened their minds to the rational possibility of participating? Ultimately at issue is whether the manner in which the information is provided can sway participants to make a decision that is against their best interest. The mere risk of this likely results in a scenario in which investigators consenting participants to research LPs would be advised to highlight both the proportion who do and the proportion who do not experience adverse events (32). Here, we have demonstrated that frame may matter, and this principle may be instructive, especially in the setting of developing recruitment and educational materials.

Although we hypothesized that frame effects would be most profound in participants with neutral pre-intervention attitudes toward LP, we did not observe a differential effect of framing by pre-intervention willingness in our models. In exploratory CART analyses, however, we did observe that pre-intervention willingness was among the strongest predictors of whether participants changed their response in our registry, more so than demographic characteristics, medical or family history, or even a measure of research attitudes. In the CART model, participants most strongly opposed to considering research LP were least likely to change their response. Among participants who reported being moderately unlikely to enroll in studies involving an LP, those with a family history of AD were more likely to reconsider their agreement. Thus, education may be most effective in those with less negative attitudes and those with a personal connection to the research topic.

Repeatedly inviting participants in longitudinal studies to consider LP may improve rates of participation in optional biomarker research. Even without intervention, a small proportion of participants (10%) in this study indicated a willingness to consider research LP based solely on being asked again (i.e., prior to educational intervention). Our data do not explain why this change occurred in these subjects, but it is possible that the engagement that accompanies enrollment in the registry “nudged” some subjects to increase their willingness to participate in additional research (33).

This study has several limitations. While the results are promising, it is unclear whether a shift in willingness to consider research LP will translate into actual behaviors (i.e., participation in CSF research). We note, however, that receptiveness to be contacted about studies is more tangible than purely hypothetical responses. As noted above, clinicians consenting participants and performing the research LP may not be comfortable exclusively using gain framed education (i.e., focused only on the proportion of subjects free of adverse events). Nonetheless, these findings may be instructive to the development of recruitment materials for studies requiring LP. We adopted AE rates from a single, somewhat older study (5) when developing our educational materials. It is possible that citing studies with higher rates (3, 4) could have produced different results. Further limiting the generalizability of our findings is the overrepresentation of white, college educated and presumably technologically savvy participants (due to their enrollment in an online registry) in our sample. There was no control group to permit rigorous examinations of the education effect. Nevertheless, information about pre-intervention willingness and pre- and post-intervention perceptions of the LP are informative about the value of education. Lastly, randomization in this study was assigned prior to asking about willingness to undergo an LP. A small but measurable proportion of participants who previously reported that they were unwilling to be contacted about studies involving LP indicated in this study that they would be either moderately or extremely likely to enroll in a study with LP. Exclusion of these subjects from the randomized study would have ensured we were investigating a population still unwilling to consider LP at baseline.

In conclusion, these results indicate that inexpensive, low burden educational interventions may increase willingness to participate in research involving LP. We demonstrate the principle that the manner in which information is presented, framing, may impact willingness to consider participation. Future research should examine whether educational interventions are equally effective in diverse populations and whether they can increase participation in specific studies that require LP.

## Data Availability Statement

The raw data supporting the conclusions of this article will be made available by the authors, without undue reservation.

## Ethics Statement

The studies involving human participants were reviewed and approved by University of California Irvine, Institutional Review Board. Written informed consent for participation was not required for this study in accordance with the national legislation and the institutional requirements.

## Author Contributions

MW was responsible for the design and conceptualization of the study, data collection, and drafting of the manuscript for intellectual content. OB and CS were responsible for data analysis and drafting and revision of the manuscript. VL, CC, SS, and DH were responsible for revision of the manuscript for intellectual content. DG and JG were responsible for the design and conceptualization of the study, data collection, drafting, and revision of the manuscript for intellectual content. All authors contributed to the article and approved the submitted version.

## Conflict of Interest

The authors declare that the research was conducted in the absence of any commercial or financial relationships that could be construed as a potential conflict of interest.
